# Isolation of Exosomes from Blood Plasma: Qualitative and Quantitative Comparison of Ultracentrifugation and Size Exclusion Chromatography Methods

**DOI:** 10.1371/journal.pone.0145686

**Published:** 2015-12-21

**Authors:** Tamás Baranyai, Kata Herczeg, Zsófia Onódi, István Voszka, Károly Módos, Nikolett Marton, György Nagy, Imre Mäger, Matthew J. Wood, Samir El Andaloussi, Zoltán Pálinkás, Vikas Kumar, Péter Nagy, Ágnes Kittel, Edit Irén Buzás, Péter Ferdinandy, Zoltán Giricz

**Affiliations:** 1 Department of Pharmacology and Pharmacotherapy, Semmelweis University, Budapest, Hungary; 2 Department of Biophysics and Radiation Biology, Semmelweis University, Budapest, Hungary; 3 Department of Genetics, Cell- and Immunobiology, Semmelweis University, Budapest, Hungary; 4 Department of Rheumatology, Polyclinic of the Hospitaller Brothers of St John of God, Budapest, Hungary; 5 Department of Physiology, Anatomy and Genetics, University of Oxford, Oxford, United Kingdom; 6 Institute of Technology, University of Tartu, Tartu, Estonia; 7 Department of Laboratory Medicine, Karolinska Institutet, Stockholm, Sweden; 8 Department of Molecular Immunology and Toxicology, National Institute of Oncology, Budapest, Hungary; 9 Centre for Cellular and Molecular Platforms, NCBS, Bangalore, India; 10 Department of Pharmacology, Institute of Experimental Medicine, Hungarian Academy of Sciences, Budapest, Hungary; 11 Pharmahungary Group, Budapest, Hungary; Tecnologico de Monterrey, MEXICO

## Abstract

**Background:**

Exosomes are emerging targets for biomedical research. However, suitable methods for the isolation of blood plasma-derived exosomes without impurities have not yet been described.

**Aim:**

Therefore, we investigated the efficiency and purity of exosomes isolated with potentially suitable methods; differential ultracentrifugation (UC) and size exclusion chromatography (SEC).

**Methods and Results:**

Exosomes were isolated from rat and human blood plasma by various UC and SEC conditions. Efficiency was investigated at serial UC of the supernatant, while in case of SEC by comparing the content of exosomal markers of various fractions. Purity was assessed based on the presence of albumin. We found that the diameter of the majority of isolated particles fell into the size range of exosomes, however, albumin was also present in the preparations, when 1h UC at 4°C was applied. Furthermore, with this method only a minor fraction of total exosomes could be isolated from blood as deduced from the constant amount of exosomal markers CD63 and TSG101 detected after serial UC of rat blood plasma samples. By using UC for longer time or with shorter sedimentation distance at 4°C, or UC performed at 37°C, exosomal yield increased, but albumin impurity was still observed in the isolates, as assessed by transmission electron microscopy, dynamic light scattering and immunoblotting against CD63, TSG101 and albumin. Efficiency and purity were not different in case of using further diluted samples. By using SEC with different columns, we have found that although a minor fraction of exosomes can be isolated without significant albumin content on Sepharose CL-4B or Sephacryl S-400 columns, but not on Sepharose 2B columns, the majority of exosomes co-eluted with albumin.

**Conclusion:**

Here we show that it is feasible to isolate exosomes from blood plasma by SEC without significant albumin contamination albeit with low vesicle yield.

## Introduction

Extracellular vesicles (EVs) are small membrane-enclosed structures with various functions and different origins. So far, three major types of extracellular vesicles have been characterized extensively: apoptotic bodies (>1000 nm), microvesicles (100–1000 nm), and exosomes (30–100 nm). Apoptotic bodies are released by apoptotic cells, microvesicles are shed from plasma membrane, while exosomes are secreted through the endosome-multivesicular body complex by most cell types (*e*.*g*., reticulocytes [1], dendritic cells [2], endothelial cells [3], tumor cells [4], or cardiomyocytes [5]; see for review: [6]). A continuously growing number of reports indicate that EVs, particularly exosomes convey information to neighboring or remote cells by delivering RNAs and proteins and thus affecting various physiological and pathological signaling pathways [7–10]. Furthermore, the therapeutic use of EVs has been investigated in a growing number of publications [11]. However, despite the high interest in the field, standardization and further characterization of separation techniques used in EV studies are yet to be performed.

Methods to isolate an EV subpopulation, exosomes, are subjects of constant debate (see for reviews [6, 12, 13]). For investigations on exosomes isolated from cell culture supernatants, methods that involve ultracentrifugation (UC) are most commonly used [13, 14]. In contrast to exosomes isolated from conditioned media, neither the efficiency nor the purity of exosome isolates from biological fluid samples are well-established. However, for diagnostic applications, isolation of exosomes from blood plasma may also be necessary, as blood is the most widely used source for biomarker research. Reproducible isolation of biologically active, intact exosomes with high efficiency and without impurities from as complex matrices as plasma is technically challenging and has not been standardized. Exosome isolation from plasma, as from cell cultures, is often performed with UC-based methods, although concerns about efficiency and purity have been raised [15]. Recently, it has been proposed that with size exclusion chromatography (SEC) exosome isolation from blood plasma can be performed without significant impurities [16–19]. However, the efficiency of exosome isolation by SEC is still debated. Furthermore, various SEC matrices (e.g., Sepharose 2B, Sephacryl S-400) have been utilized for exosome isolation, but so far comparative analyses on their quantitative and qualitative performances have not been reported [16–18].

Therefore, here we aimed to investigate the efficiency of UC and SEC-based methods of exosome isolation from blood plasma, and purity of the resulting isolates. We found that exosomes can be isolated by SEC without significant albumin contamination albeit at a low yield.

## Materials and Methods

This investigation conforms to the Guide for the Care and Use of Laboratory Animals published by the US National Institutes of Health (NIH publication No. 85–23, revised 1996), to the EU Directive (2010/63/EU) and was approved by the animal ethics committee of the Semmelweis University, Budapest, Hungary. Before blood collection from human individuals, written informed consent was obtained. In these experiments we followed the regulations of Helsinki Declaration (1975), and investigations were supervised and specifically approved by the Hungarian Scientific and Research Ethics Committee and the Institutional Review Board of the Polyclinic of the Hospitaller Brothers of St John of God, Budapest (Approval number: 22244/2013/EKU(280/2013)).

### Preparation of blood plasma

Whole blood was collected from male Wistar rats (250–300 g; 45–50 days old) from the abdominal aorta into Anticoagulant Citrate Dextrose-A (ACD-A)-containing tube (Greiner, Mosonmagyaróvár, Hungary) [20]. Venous blood samples (9 mL) were collected from the cubital vein of four healthy human volunteers (3 men and 1 woman) without any known acute or chronic diseases into ACD-A-containing tube via BD Vacutainer^®^ blood collection system (Greiner, Mosonmagyaróvár, Hungary). Human subjects were between 25 and 45 years old and were not taking any medication at the time of sampling. Cellular components were eliminated with centrifugation (two times 2,500×g, 4°C, 15 min). Supernatant (*i*.*e*., platelet-free plasma) was diluted to 2× with phosphate-buffered saline (PBS), and was filtered through a 0.8 μm filter (Merck, Darmstadt, Germany) by hydrostatic pressure to remove remaining platelets and apoptotic bodies.

### Isolation of exosomes from blood plasma with ultracentrifugation

Seven mL of 0.8 μm filtered and 2× PBS-diluted platelet-free plasma samples were centrifuged at 13,200×g and 4°C for 22 min to remove microvesicles. The supernatant was filtered twice through 0.22 μm filters (pre-cleared plasma). Exosomes were pelleted with UC at 120,000×g in an MLA-55 fixed-angle rotor (Beckman Coulter, Brea, CA, US) with various conditions. Either 7 mL pre-cleared plasma was centrifuged for (a) 1h, (b) 3h, (c) 6h, (d) 14h at 4°C, or (e) for 1h at 37°C (supplemented with protease inhibitor [Amresco, Solon, OH, US]). Furthermore, in experiment (f) 7 mL 5× diluted pre-cleared plasma (ultimately 10-fold dilution, since platelet-free plasma was 2× diluted before) was centrifuged for 1h at 4°C, and in experiment (g) 1 mL pre-cleared plasma was centrifuged for 1h at 4°C. In case of experiments (a), (e), (f), and (g), supernatants after ultracentrifugation were repeatedly ultracentrifuged with the same conditions. This procedure was repeated three times. Pellets were washed once with PBS and centrifuged at 120,000×g, 4°C for 1h in case of samples that were centrifuged for 1h in the previous step or for 2h in experiments (b), (c), and (d). Samples were used fresh, since our experiments indicated that integrity of exosomes is compromised when stored for 4 weeks or longer (Fig A-C in S1 File)

### Isolation of exosomes from blood plasma with SEC

SEC was performed as described previously with minor modifications [16]. Briefly, 0.5 mL of 0.8 μm-filtered blood plasma sample was diluted 2 fold with PBS and loaded onto gravity-eluted columns of various matrices (Sepharose 2B [Sigma, St. Louis, MO, US], Sepharose CL-4B [Sigma, St. Louis, MO, US], or Sephacryl S-400 [GE Healthcare, Little Chalfont, UK]) with 10 mL bed volume. One mL fractions were eluted with PBS. First fractions were collected right after the blood plasma has been loaded, and a total of 10 mL eluate was collected. For transmission electron microscopy, fractions were concentrated to 110 μL on 10-kDa molecular weight cut-off Amicon Ultra spin filters (Merck, Darmstadt, Germany).

In a separate experiment, rat (1 mL) plasma sample was loaded onto a 120 mL bed volume Sephacryl S-400 column (GE Healthcare, Little Chalfont, UK) connected to an ÄKTA pure 25 L (GE Healthcare, Little Chalfont, UK) liquid chromatography system equipped with an UV/VIS spectrophotometer trained at 280 nm. Samples were eluted with PBS at 0.5 mL/min flow rate and collected into 2 mL fractions during 0–0.45 column volumes and into 5 mL fractions during 0.45–1.1 column volume. Particle concentration and size distribution was measured at each fraction using Nanoparticle Tracking Analysis NS500 instrument (NTA) (Malvern, Worcestershire, UK), as described below. Fractions were combined into 5 pooled fractions based on the A280 chromatogram and concentrated to 250 μL using 10-kDa molecular weight cut-off Amicon Ultra spin filters (Merck, Darmstadt, Germany).

### Transmission electron microscopy (TEM)

In experiments with UC, exosomes were re-pelleted in MLS-50 swinging-bucket rotor (Beckman Coulter, Brea, CA, US). Formalin (4%) was layered carefully onto the pellets. After formalin was washed out, pellets were post-fixed with 1% osmium tetroxide (OsO_4_) for 20–30 min, and block stained with 1% uranyl acetate (in 50% ethanol) for 20–30 min. Then, pellets were dehydrated by graded ethanol for 5 min in 70%, 90%, 96%, and 3×5 min in 100% ethanol. Pelleted samples were embedded in Taab 812 (Taab Laboratories, Aldermaston, UK). Then ultrathin sections were prepared.

Exosome samples obtained by SEC were fixed by buffered 1% OsO_4_ solution in 1:1 for 30 min. Formvar-coated TEM grids were placed on top of 5–10 μL drops of these fixative-containing samples for 20 min, then grids were consecutively transferred to drops of distillated water (3x5 min), 1% uranyl-acetate in 50% alcohol for contrast staining for 15 min and finally to drops of distilled water for 3x5 min. Grids carefully removed from the last drop of water were air dried and analyzed.

Prepared samples were analyzed under Hitachi 7100 electron microscope equipped by Veleta, a 2k×2k MegaPixel side-mounted TEM CCD camera (Olympus, Tokyo, Japan). Contrast and brightness of electron micrographs were edited in Adobe Photoshop CS3 (Adobe Systems Incorporated, San Jose, CA, US).

### Dynamic light scattering (DLS) and NTA

Size distribution of exosomes was measured by DLS measurements by using an ALV goniometer with a Melles Griot diode-pumped solid-state laser at 457.5 nm wavelength (type: 58 BLD 301). The intensity of the scattered light was measured at 90° and the autocorrelation function was calculated using an IBM PC-based data acquisition system. Particle size distributions were determined by the maximum entropy method. The diameter of the particles was calculated using sphere approximation.

Concentration and size distribution profile of the particles isolated by the 120 mL Sephacryl S-400 column was evaluated using a NanoSight NS500 instrument (Malvern, Worcestershire, UK) and NTA 2.3 software. Videos were recorded at camera level 15. The following post acquisition settings were selected: minimum detection threshold 5, automatic blur, and automatic minimum expected particle size. Samples were diluted 1:1000–1:10000 in PBS to achieve measured particle concentration 5–15x10^8^/mL. For each sample, three 30 sec videos were recorded and analyzed in the batch processing mode.

### Western blot

In case of samples obtained after UC, pellets were homogenized with 1× radio immunoprecipitation assay (RIPA; Cell Signaling Technology, Danvers, MA, US) containing protease inhibitor (Amresco, Solon, OH, US). In case of SEC, for equal protein loading, proteins were precipitated with trichloroacetic acid (20% final concentration), and precipitates were homogenized with 1× RIPA (Cell Signaling Technology, Danvers, MA, US) containing protease inhibitor (Amresco, Solon, OH, US). For equal volume loading measurements, nonhomogenized samples were used. Protein concentration of the homogenates was measured by bicinchoninic acid assay kit (Thermo Scientific, Waltham, MA). Equal volume or equal protein amount of each sample was mixed with reducing Laemmli-buffer and was loaded on 4–20% Tris-glycine sodium dodecyl sulfate-polyacrylamide gels (Bio-Rad, Hercules, CA, US), and electrophoresed. Proteins were transferred to either polyvinylidene difluoride or nitrocellulose membrane (Bio-Rad, Hercules, CA, US). Membranes were blocked in 5% non-fat milk (Bio-Rad, Hercules, CA, US) in Tris-buffered saline supplemented with 0.05% Tween-20 (TBS-T) for 2h, and then were incubated with primary antibodies (anti-CD63 [1:1000; Abcam, Cambridge, UK, ab108950], anti-TSG101 [1:1000; Abcam, Cambridge, UK, ab83] or anti-albumin [1:20000; Santa Cruz Biotechnology, Dallas, TX, US, sc-271605]) for 16 h at 4°C. After 3 washes in TBS-T, membranes were incubated with corresponding HRP-conjugated secondary antibodies for 2h at room temperature and washed in TBS-T. Signals were visualized after incubation with enhanced chemiluminescence kit (Bio-Rad, Hercules, CA, US) by Chemidoc XRS+ (Bio-Rad, Hercules, CA, US).

Equal volume loading was applied to approximate the overall efficiency of exosome isolation, since it compares the exosome concentration between fractions, however, it does not allow quantification of sample purity. Whereas equal amount of protein was loaded to evaluate the purity of isolates, since it indicates the relative amount of proteins present in the isolates.

### Statistics

Data are expressed as *mean ± standard error of mean*. *Student’s t-test* was used for the comparison of two groups. In case of multiple groups, *one-way analysis of variance* was performed with Fisher’s least significant difference (LSD) as a *post hoc* test. Statistical significance was accepted if *p<0*.*05*.

## Results

### Yield of exosome isolation with UC and purity of isolates

**Fig 1:**
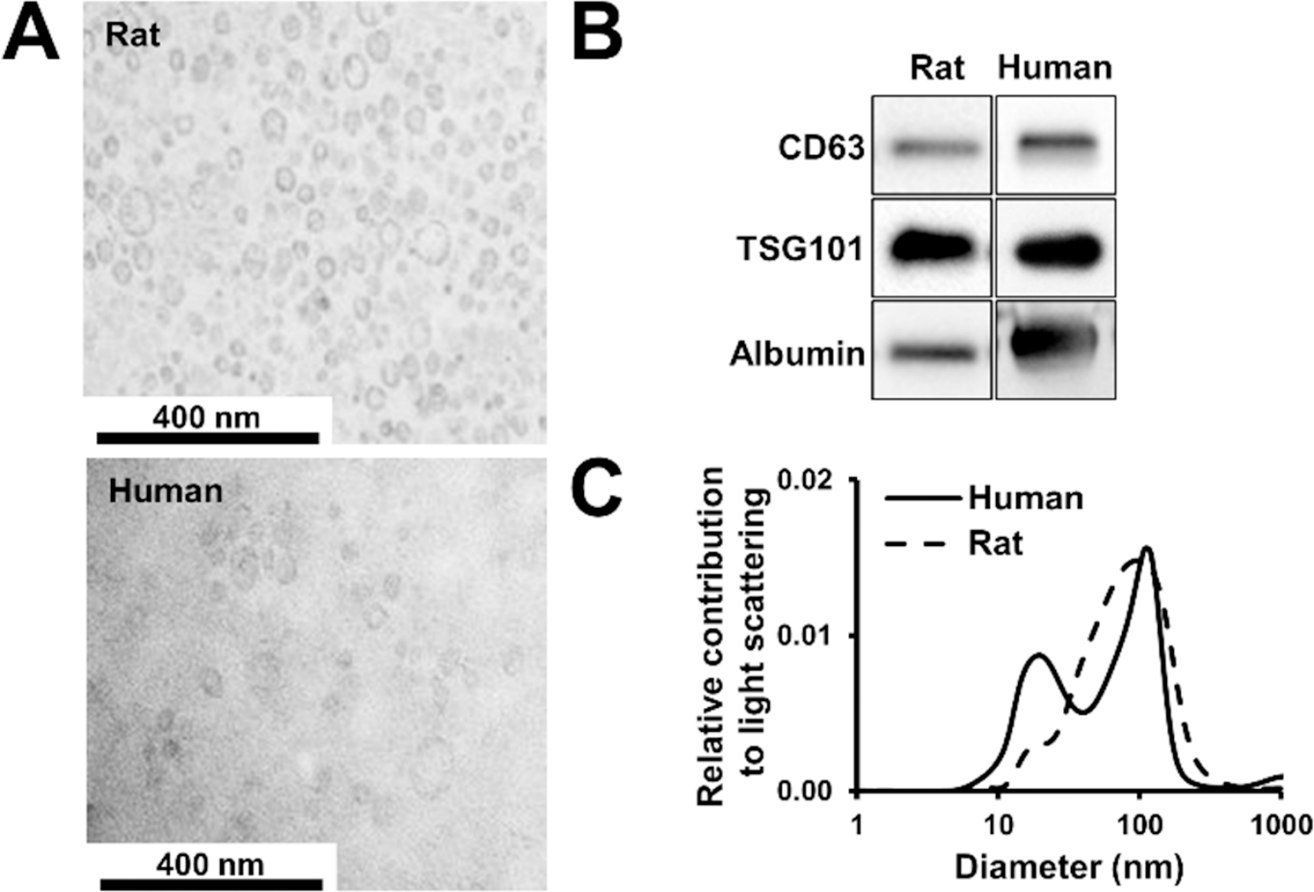
Exosomes can be isolated with 1h ultracentrifugation method from blood plasma. (A) Transmission electron microscopy images of exosome isolates from rat and human blood plasma. (B) CD63, TSG101 and albumin content of the rat and human exosomal isolates as evaluated with Western blot. (C) Size distribution of particles isolated from rat and human blood plasma analyzed with dynamic light scattering (averages of n = 3–4).

Rat and human exosomes were isolated by differential centrifugation and filtration as described above. After 1h of UC (*i*.*e*., experiment (a)), structurally intact exosomes could be detected by TEM (Fig 1A), which displayed exosomal markers (CD63 and TSG101, Fig 1B). To assess the amount of impurities in isolates, albumin was chosen as a characteristic marker, since it is the most abundant plasma protein. In addition, liquid chromatography–mass spectrometry (LC-MS)-based proteomics experiments revealed that albumin as well as a number of other plasma proteins are overrepresented in the UC-derived preparations (Table A in S1 File). Accordingly, UC- derived isolates contained a significant amount of albumin as assessed by Western blots (Fig 1B). DLS analysis revealed that the diameter of the majority of particles were between 30–100 nm in an aqueous environment after 1h UC (Fig 1C).

**Fig 2:**
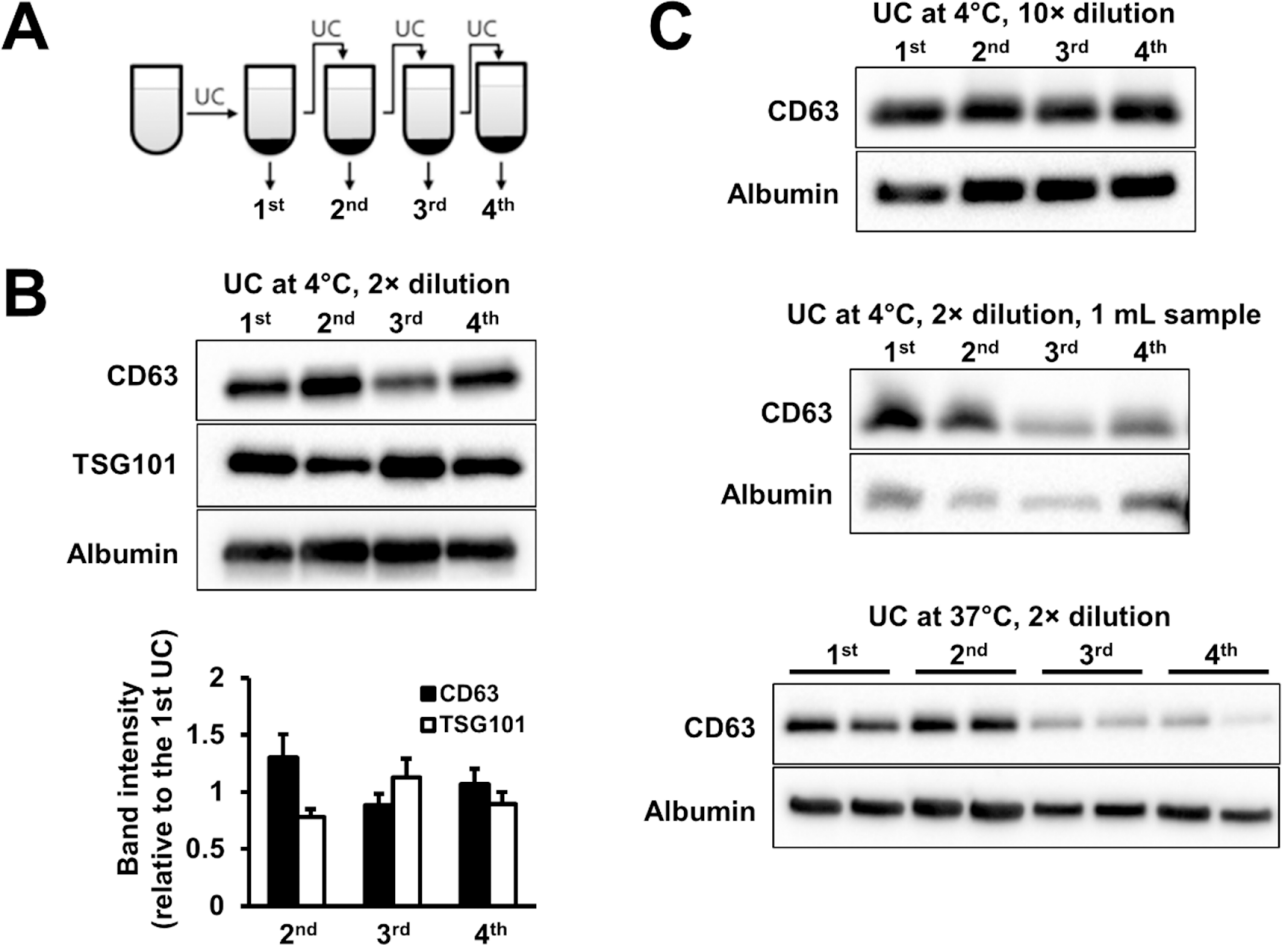
Efficiency of ultracentrifugation (UC) methods under various conditions. (A) Scheme of serial UC of the supernatant. Exosomal marker CD63 and TSG101 and albumin content of exosomal isolates after serial UC of the supernatant with 1h UC at 4°C (B; n = 3; p>0.05), with 10-fold dilution at 4°C (C, top), with smaller volume of loaded sample at 4°C (C, middle) and with 1h UC at 37°C (C, bottom) as evaluated with Western blot.

To assess efficiency of exosome isolation from blood plasma by UC, after each UC the remaining supernatant was re-ultracentrifuged four times consecutively, and the pellets obtained from each centrifugation round were analysed by Western blotting (Fig 2A). The amount of CD63 and TSG101 detected by Western blotting in the pelleted exosome isolates after repeated 1h UC of the samples was comparable instead of showing a decreasing trend (experiment (a)). This indicates that only the minority of exosomes could be isolated with 1h UC (Fig 2B). Furthermore, albumin was present in all subsequent isolates. Then we analyzed the efficiency of UC while modulating various parameters (such as sample viscosity and sedimentation distance) which might potentially influence the efficiency of the exosome isolation. UC of 10-fold diluted blood plasma (experiment (f)) or UC with smaller sedimentation distance (1 mL sample; experiment (g)) resulted in approximately the same yield as UC of 7 mL 2-fold diluted plasma. Albumin was also detectable after each UC cycle in these experiments (Fig 2C). The efficiency of UC of approximately 7 mL plasma at 37°C (experiment (e)), however, proved to be higher than that of the UC performed at 4°C since after each UC cycle less CD63 was present in the consecutive pelleted exosome isolates. However, albumin content of the isolates was still significant (Fig 2C).

**Fig 3:**
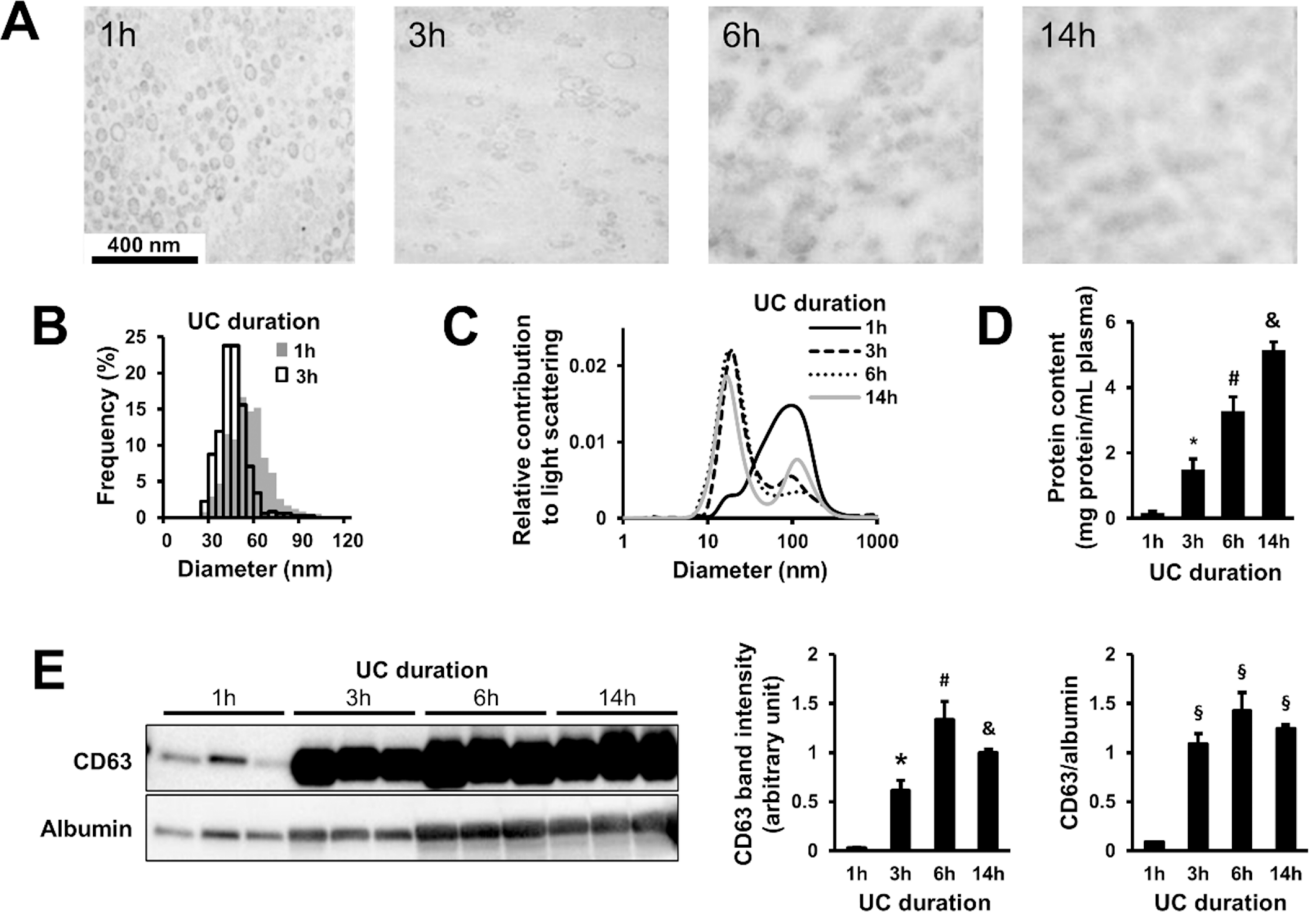
The effect of various ultracentrifugation (UC) duration on the exosomal yield and purity. (A) Transmission electron microscopy images of rat exosomes isolated with 1, 3, 6 or 14h UC period. (B) Size distribution of rat exosomes based on transmission electron microscopy image analysis (1h UC: n = 2,440; 3h UC: n = 353). (C) Size distribution of particles isolated with different UC duration evaluated with dynamic light scattering (averages of n = 3). (D) Protein concentration of exosome homogenates isolated with different UC duration as assessed with bicinchoninic acid assay (n = 3–7; *, #, &: p<0.05 vs. other three groups). (E) CD63 and albumin content of exosome isolates with different UC length (n = 3; *, #, &: p<0.05 vs. corresponding other three groups; §: p<0.05 vs. 1h).

We also isolated exosomes with various duration of UC, i.e. 1h, 3h, 6h and 14h (experiment (a), (b), (c) and (d), respectively). Exosomes could be visualized with TEM only in isolates which were centrifuged for 1h or 3h (Fig 3A and 3B). Exosomes in 1h and 3h UC pellets appeared to be structurally intact. Some vesicular structures could also be identified after 6h and 14h UC, although a high amount of amorphous material obscured them. DLS analysis established that 1h UC samples contained predominantly exosomes with expected size range, however, after 3h, 6h and 14h of UC a smaller proportion of particles fell into the exosomal range (Fig 3C). The predominant particle population was significantly larger in isolates centrifuged for 1h (mode diameter: 98.49 nm; n = 3) than in those of 3h, 6h and 14h (18 nm, 19 nm and 18 nm, respectively; n = 3–5; p<0.05 vs. 1h UC; Fig 3C). We observed that the protein content of the samples was increased with the duration of UC (Fig 3D). Western blot analysis was performed to estimate the quantity and purity of isolated exosomes. In case of longer UC periods, significantly higher CD63 signal was detected, and CD63/albumin ratio was simultaneously elevated as well (Fig 3E).

### Yield of exosome isolation with SEC and purity of isolates

**Fig 4:**
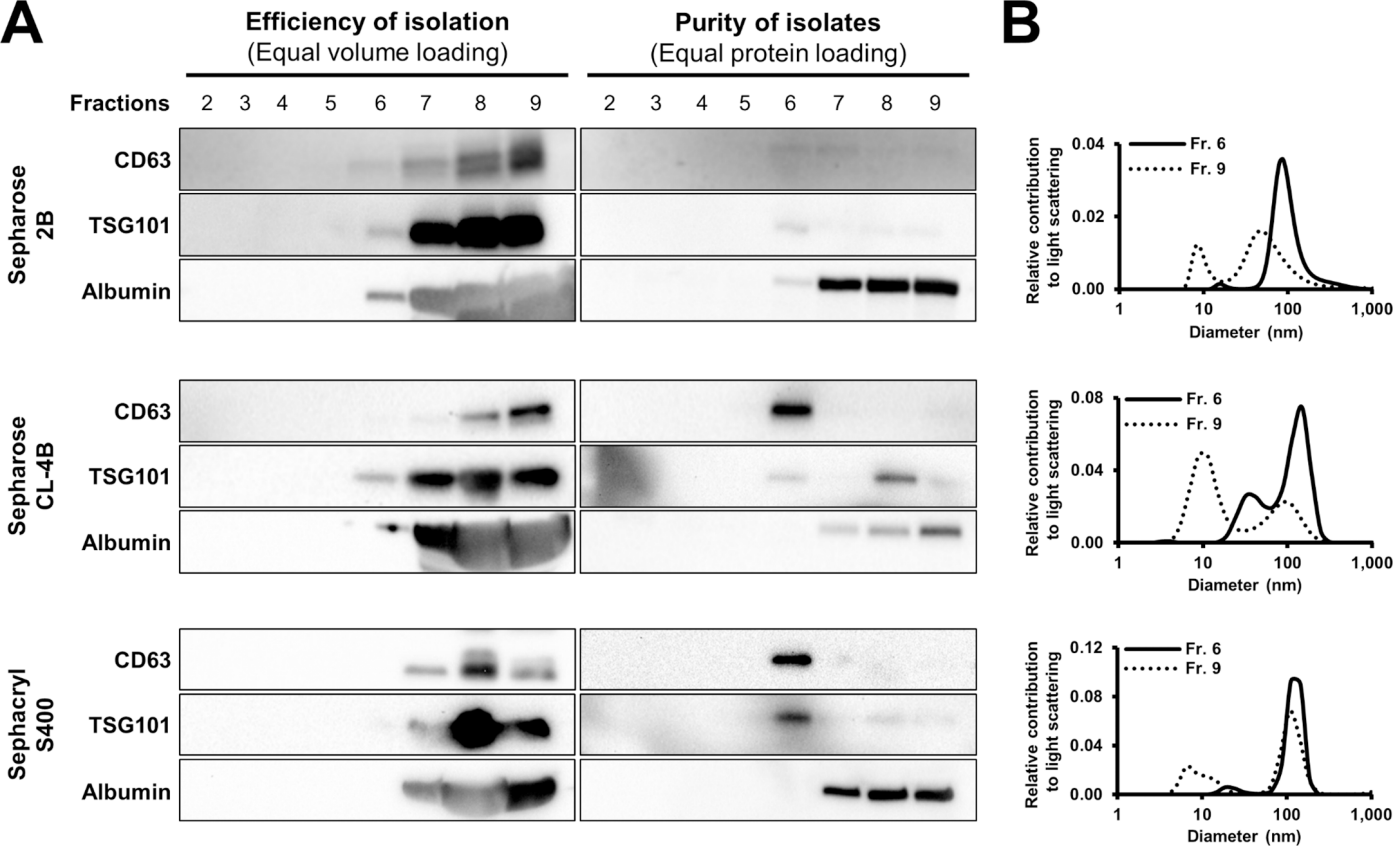
Efficiency and selectivity of size exclusion chromatography (SEC) on exosome isolation performed with various matrices. (A) CD63, TSG101 and albumin content of different fractions collected during SEC on Sepharose 2B (top), Sepharose CL-4B (middle) and Sephacryl S-400 (bottom) columns with equal volumes (left column) or equal protein amounts of fractions (right column) loaded for Western blot. (B) Size distribution of particles isolated with various SEC matrices evaluated with dynamic light scattering. Sepharose 2B (top), Sepharose CL-4B (middle) and Sephacryl S-400 (bottom) columns.

Several SEC media (including Sepharose 2B, Sepharose CL-4B Sephacryl S-400) were evaluated for the isolation of exosomes from blood plasma. To assess efficiency of the isolation, equal volumes of SEC fractions were analyzed by Western blots, while purity is tested by loading equal amount of protein. In case of Sepharose 2B, CD63 and TSG101 were detected predominantly in fractions 6–7, but albumin was also present in these fractions (Fig 4A). However, fraction 6 from Sepharose CL-4B and Sephacryl S-400 columns presented exclusively CD63 and TSG101 signals without significant albumin impurity (Fig 4A). By loading equal volumes of SEC fractions, the vast majority of CD63 and TSG101 was co-eluting with albumin in case of every medium, which indicates that separation efficiency was low, approximately below 1% (Fig 4A). Similarly, DLS measurements demonstrated that early fractions (e.g. fraction 6) contained particles with a modal diameter corresponding to exosomes (2B: 87 nm; CL-4B: 149 nm; S-400: 145 nm). Meanwhile in case of later fractions (*e*.*g*., fraction 9), a population of particles with a significantly smaller diameter (<30 nm) was present (Fig 4B).

**Fig 5:**
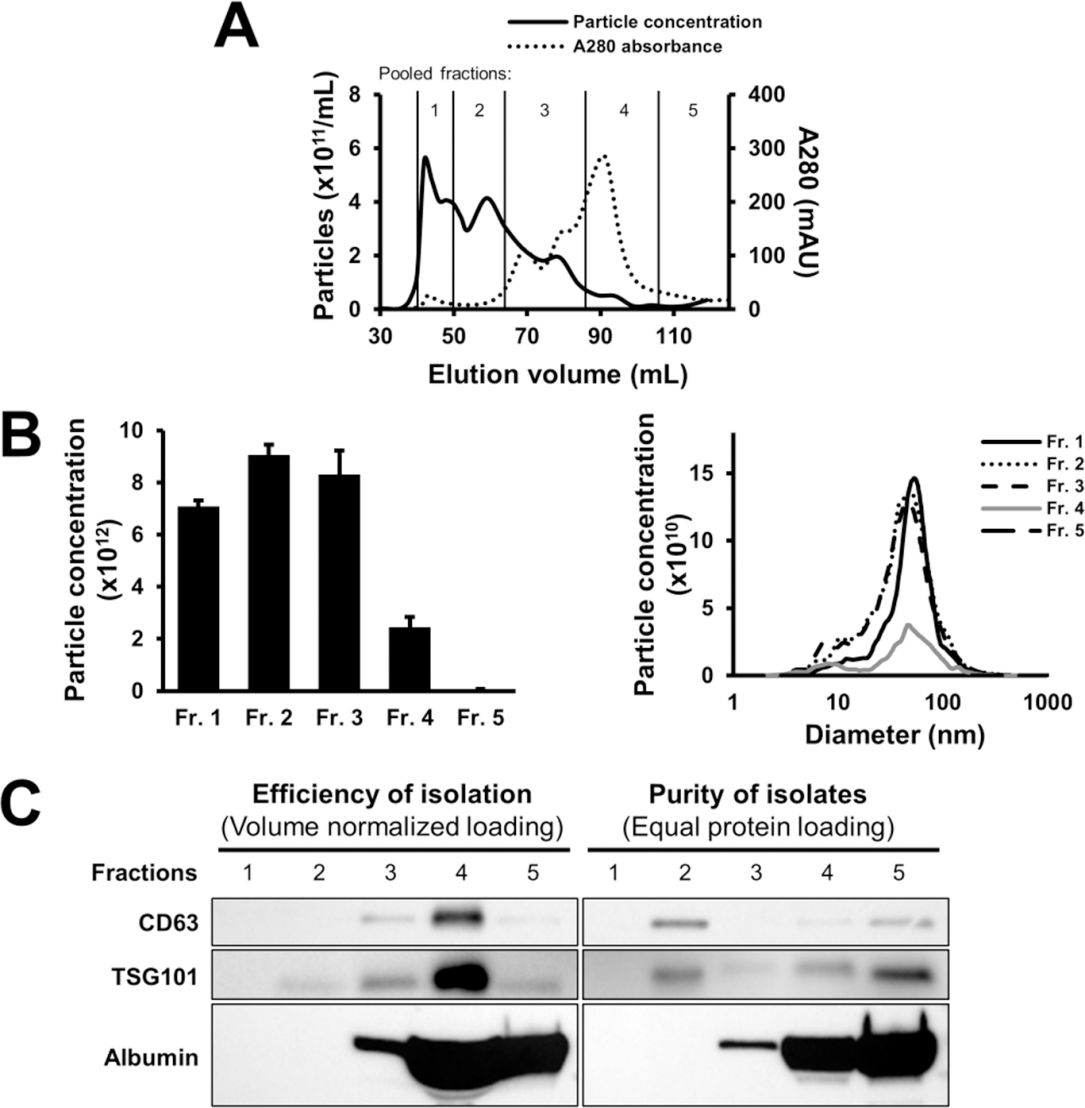
Size exclusion chromatography isolation of exosomes from rat blood plasma on a large-scale Sephacryl S-400 column. (A) Particle concentration and protein content (absorbance at 280 nm) of eluted fractions. (B) Concentration and size distribution of particles in pooled fractions by nanotracking analysis. (C) CD63, TSG101 and albumin content of pooled

We also investigated the efficiency and purity of exosomes isolated with a large-scale Sephacryl S-400 column from rat plasma. According to NTA measurements, the majority of the particles with exosomal diameter was eluted in the early pooled fractions (*i*.*e*., pooled fractions 1, 2 and 3) before the major protein peak (Fig 5A and 5B). Exosomes without significant albumin impurity were detected in the pooled fraction 2. However, similarly to 10 mL SEC columns, the majority of CD63 and TSG101 signals co-eluted with albumin (Fig 5C). It has to be noted that although NTA indicated that pooled fractions 1–3 contained the majority of particles, this finding did not correlate with Western blot results on exosome markers CD63 and TSG101, which showed that the majority of exosomes was contained by pooled fraction 4.

## Discussion

Here we evidenced that exosomes can be isolated by SEC from blood plasma without significant amount of albumin but not by UC, and that only a minority of exosomes can be isolated by SEC or UC from the blood.

Efficiency of exosome isolation methods and the purity of isolates have not been thoroughly previously characterized for blood plasma. Although UC has been the most commonly used method to isolate exosomes, here we found that only a minority of exosomes could be isolated from the blood plasma. This is in agreement with previous reports, where the efficiency of exosome isolation from highly viscous cell culture media with UC was less than 5% [15]. One might speculate that modifying isolation parameters, such as viscosity, sedimentation distance and temperature, or the prolongation of UC time might increase the yield of exosomes. Indeed, performing UC at 37°C (experiment (e)) or with increased duration, *e*.*g*., 3–14h (experiment (a)-(d)), resulted in higher exosomal yield. Similarly, Shelke *et al*. evidenced a high efficiency of extended UC as exosomal RNA was not detectable in bovine serum after 18h UC [21]. Nevertheless, it should be mentioned that the determination of efficiency in our and abovementioned studies is only an approximation, since, to date, there is no methodology providing exact quantitative analysis of EVs that meets most criteria, *e*.*g*., selectivity, reproducibility and robustness. To assess the presence of impurities, here we demonstrated that albumin content of UC-derived isolates was significant. This is in agreement with previous findings where the majority of proteins, including albumin, detected in UC-isolated exosomal preparations, was unrelated to the conventional exosomal proteome [22]. Since albumin is the major soluble plasma protein and a predominant component of the isolates, we conclude that UC is not an optimal method to obtain a pure exosomal preparation *i*.*e*. as needed for *in vivo* experimentation or for analytical assays (*e*.*g*., proteomics or RNA analysis), however, exosomes isolated by UC might be appropriate for analyses where contaminating materials do not interfere with measurements. By immunoblotting here we demonstrated that the purity of isolates after 3h UC was superior to the 1h UC. In contrast, DLS analysis revealed that the mode diameter of the predominant particle population in 3h UC isolates was 18 nm indicating that the majority of the particles might be protein aggregates rather than vesicles. This was further confirmed by TEM, where significant amount of amorphous substance obscured the vesicular structures. Nevertheless, these results highlight that experiments with exosomal isolates obtained with UC require careful designing and well-defined controls, such as applying detergent treatment [23].

Recently, SEC was introduced as a novel method to purify exosomes from blood plasma [16, 17, 24], but efficiency of isolation and comparative studies on different SEC media have not been performed. Here we showed that SEC on 10 mL Sepharose CL-4B or Sephacryl S-400 columns provided exosome isolates free from albumin, which is in agreement with other’s findings of various media [16, 17, 24]. Interestingly, in our experiments, the purity of exosomal isolates obtained by the most commonly used media Sepharose 2B was inferior to other investigated media. This observation highlights that systematic analysis and optimization of SEC-based assays is still warranted. Here, we also found that SEC performed on 10 mL columns, is not an effective method for the isolation of exosomes, its efficiency is comparable to those of the UC-based methods. This finding contradicts recent results which show that commercially available 10 mL ExoSpin or qEV columns separate exosomes from soluble contaminants with high efficiency [19, 24]. Although these publications evidence a good separation of vesicular markers from numerous soluble contaminants, efficiency was not assessed in comparison with the input quantities. Discrepancies between these studies and our results might be attributed to the different methods with which efficiency was estimated. Here we relied on the detection of exosomal markers by Western blot, while others prefer particle counting assays such as NTA or resistive pulse sensing, the reliability of which is under constant debate in the range of particles with a diameter of less than 100 nm, *i*.*e*., exosomes [25]. This phenomenon was also evidenced in this study, as NTA and Western blot results did not correlate well in case of the large-scale SEC experiment. Nevertheless, the use of SEC to obtain high-purity exosomes is well confirmed [16, 18, 24]. However, it is plausible that adjusting column characteristics, such as volume or type of medium, or optimizing the applied sample amount might improve yields of SEC methods. In this regard, here we show that SEC performed on a 120 mL Sephacryl S-400 column does not result in a significantly improved yield, suggesting that SEC may have its inherent limitations for exosome isolation from blood plasma.

Here we assessed that exosomes can be isolated by SEC without significant albumin content, however, this study has limitations. The purity of exosomes isolated by SEC was estimated by the presence of albumin alone. Although albumin is the most abundant serum protein, other soluble proteins might distribute differentially and might still be present in our preparations. In addition, we cannot exclude the possibility that serum proteins, especially albumin, and exosomes interact specifically, and therefore co-elute from SEC columns, which might explain the low efficiency of our SEC experiments.

In summary, here we demonstrate that SEC but not UC is suitable for the isolation of exosomes from blood without significant albumin contamination, although its efficiency needs to be further improved. These results suggest that experiments on exosomes isolated by UC or SEC should be evaluated carefully and by applying appropriate controls.

## Supporting Information

S1 File Supplementary methods. Storage of isolated vesicles; In gel digestion of SDS-PAGE gels stained with Colloidal Coomassie Blue; Nano-LC-MS analysis of proteins and database search. **Fig A-C:** The effect of different storage conditions on the quality of isolated exosomes. **Table A:** Most abundant proteins in exosomes isolated with 1h ultracentrifugation protocol as assessed by LC-MS based proteomics.
(DOCX)
