# Supplementary material for: Isolation of Exosomes from Blood Plasma: Qualitative and Quantitative Comparison of Ultracentrifugation and Size Exclusion Chromatography Methods
Source: PLoS One. 2015 Dec 21;10(12):e0145686. doi: 10.1371/journal.pone.0145686 (PMC4686892; doi:10.1371/journal.pone.0145686)
Supplement: S1 File — Storage of isolated vesicles; In gel digestion of SDS-PAGE gels stained with Colloidal Coomassie Blue; Nano-LC-MS analysis of proteins and database search. Fig A-C: The effect of different storage conditions on the quality of isolated exosomes. Table A: Most abundant proteins in exosomes isolated with 1h ultracentrifugation protocol as assessed by LC-MS based proteomics. (DOCX) [file pone.0145686.s001.docx]

# Supporting information:

**Supplementary methods**

*Storage of isolated vesicles*

Three and a half mL of filtered blood plasma samples were centrifuged at 13,200×g and 4°C for 22 min to remove microvesicles. The supernatant was filtered twice through 0.22 µm filters (pre-cleared plasma). Exosomes were pelleted with UC at 120,000×g in an MLA-55 fixed-angle rotor (Beckman Coulter) for 1h at 4°C. Pellets were washed once with PBS and centrifuged at 120,000×g, 4°C for 1h. To investigate characteristics of stored exosomes, exosomes were stored at either 4°C or -80°C for 4 or 8 weeks, or used freshly, within 1h.

*In gel digestion of SDS-PAGE gels stained with Colloidal Coomassie Blue*

The stained protein lane were excised into 12 bands, each band were diced into small pieces, washed twice with 50% acetonitrile in 25 mM NH_4_HCO_3_ for 10 min and dried completely using centrifugal evaporater. Before administration of trypsin, 10 mM dithiothreitol was added and incubated at 56°C for 60 min to achieve protein reduction. Following reduction, 55 mM iodoacetamide was added to alkylate the sample in the dark at room temperature for 45 min. The alkylated samples were dried again completely and digested at 37°C for 16h with 20 ng/µLporcine trypsin (dimethylated, proteomics grade, Sigma Aldrich). The digestion was quenched by adding 50% acetonitrile containing 5% formic acid. The extracted peptides were cleaned and concentrated with C18 ZipTip pipette tips (Millipore).

*Nano-LC-MS analysis of proteins and database search*

The LC-MS analysis of the tryptic peptide mixture was performed with a nanoflow HPLC system (Easy nLC II, Thermo Fisher Scientific) coupled to a LTQ XL ion trap mass spectrometer (Thermo Fisher Scientific). The injected samples were desalted on a C18 precolumn (2 cm, ID 100 µm, 5 µ m, C18-A1) and separated on a reverse phase analytical column (10 cm, ID 75 µ m, 3 µ m, C18-A2). Peptide elution from the analytical column was performed using 300 nL/min flow rate and a 40 min long gradient with increasing concentration of the organic solvent (acetonitrile containing 0.1% formic acid). Data were acquired in data dependent acquisition mode, consisting of a full scan spectrum (m/z: 400-1500), zoom scan and MS/MS spectrum of the most intense ion. During MS/MS experiments, collision energies were varied according to the mass and charge of the precursor ion.

For protein/peptide identification MS/MS data was searched against the Rattus Norvegicus database (downloaded on 28^th^ May, 2015) from RefSeq NCBI using an in-house mascot server (version 2.4) through Proteome Discoverer 1.4 software. The search was set up for full tryptic peptides with a maximum of three missed cleavage sites. Carbamidomethyl on cysteine, pyroglutamate at N terminus of peptide from both glutamine and glutamate, acetylation on N terminus of protein and oxidized methionine were included as variable modifications. The precursor mass tolerance threshold and the maximum fragment mass error was 0.5 Da. The significance threshold of the ion score was calculated based on a false discovery rate of < 1% estimated by the peptide validator node of the Proteome Discoverer software. Only proteins having minimum two high confidence peptides were selected for the analysis.

**The effect of different storage conditions on the quality of isolated exosomes.** TEM images of exosome isolates stored at 4°C or ‑80°C for 8 weeks. Black arrows indicate the preserved exosomes, while white arrows vesicular debris (**Fig A**). Size distribution of particles after various storage periods (4 and 8 weeks) analyzed with DLS (average of n=3). Proportion of vesicles within the exosomal diameter range (30-100 nm) after storage at 4°C or -80°C. n=3; *<0.05 vs. 0 week; #<0.05 vs. 4 weeks (**Fig B**). CD63, TSG101 content of exosome homogenates after various storage periods (0, 4 or 8 weeks; left). CD63 and TSG101 content of stored exosomes (right). n=3; *<0.05 vs. 0 week; #<0.05 vs. 4 weeks at 4°C (**Fig C**).


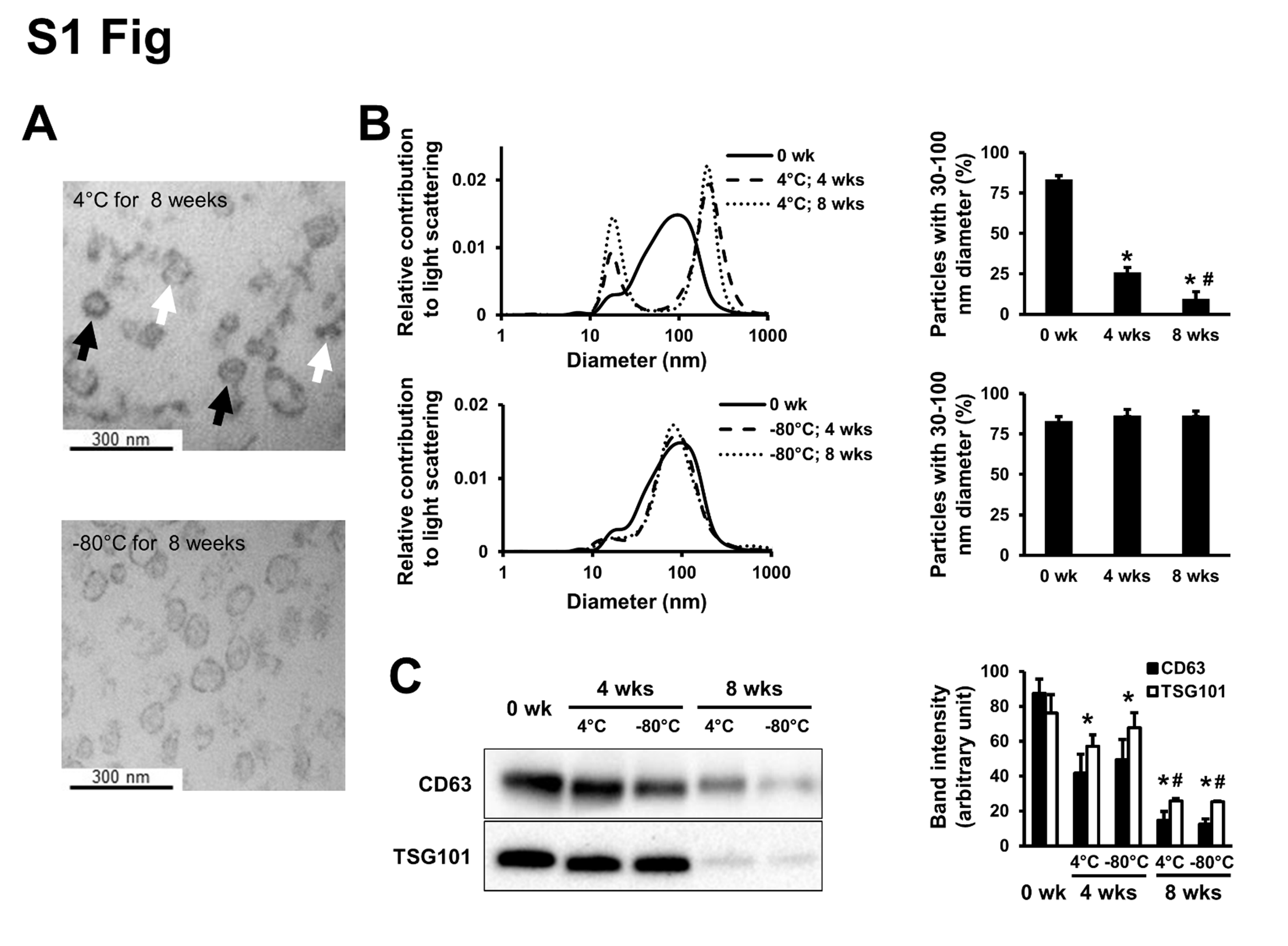


| **Rank** | **Protein** | **Accession number** | **ΣCoverage** | **Σ# Unique Peptides** |
| --- | --- | --- | --- | --- |
| 1 | fibrinogen gamma chain precursor | 61098186 | 69,57 | 20 |
| 2 | alpha-1-macroglobulin precursor | 307746876 | 62,20 | 69 |
| 3 | serum albumin precursor | 158138568 | 59,70 | 32 |
| 4 | complement C3 precursor | 158138561 | 60,01 | 68 |
| 5 | fibrinogen beta chain isoform X2 | 564336898 | 53,35 | 19 |
| 6 | serotransferrin precursor | 61556986 | 48,57 | 30 |
| 7 | apolipoprotein A-I preproprotein | 6978515 | 45,95 | 9 |
| 8 | alpha-1-antiproteinase precursor | 51036655 | 41,12 | 10 |
| 9 | fibronectin isoform X8 | 564368918 | 40,38 | 53 |
| 10 | hemoglobin subunit beta-1 | 17985949 | 47,62 | 5 |

**Table A. Most abundant proteins in exosomes isolated with 1h ultracentrifugation protocol as assessed by LC-MS based proteomics.** Based on the indicated sequence coverage and number of identified unique peptides plasma proteins are clearly overrepresented in the sample.
